# Phylogenetic Analyses of Glycosyl Hydrolase Family 6 Genes in Tunicates: Possible Horizontal Transfer

**DOI:** 10.3390/genes11080937

**Published:** 2020-08-13

**Authors:** Kun-Lung Li, Keisuke Nakashima, Jun Inoue, Noriyuki Satoh

**Affiliations:** 1 Marine Genomics Unit, Okinawa Institute of Science and Technology Graduate School, Onna, Okinawa 904–0495, Japan; keisuke@oist.jp (K.N.); norisky@oist.jp (N.S.); 2 Center for Earth Surface System Dynamics, Atmosphere and Ocean Research Institute, University of Tokyo, Kashiwa, Chiba 277-8564, Japan; jinoue@aori.u-tokyo.ac.jp

**Keywords:** Tunicates, horizontal gene transfer, cellulose synthase, Glycosyl Hydrolase Family 6, intron gain

## Abstract

Horizontal gene transfer (HGT) is the movement of genetic material between different species. Although HGT is less frequent in eukaryotes than in bacteria, several instances of HGT have apparently shaped animal evolution. One well-known example is the tunicate cellulose synthase gene, *CesA*, in which a gene, probably transferred from bacteria, greatly impacted tunicate evolution. A Glycosyl Hydrolase Family 6 (GH6) hydrolase-like domain exists at the C-terminus of tunicate CesA, but not in cellulose synthases of other organisms. The recent discovery of another GH6 hydrolase-like gene (*GH6-1*) in tunicate genomes further raises the question of how tunicates acquired GH6. To examine the probable origin of these genes, we analyzed the phylogenetic relationship of GH6 proteins in tunicates and other organisms. Our analyses show that tunicate GH6s, the *GH6-1* gene, and the GH6 part of the *CesA* gene, form two independent, monophyletic gene groups. We also compared their sequence signatures and exon splice sites. All tunicate species examined have shared splice sites in GH6-containing genes, implying ancient intron acquisitions. It is likely that the tunicate *CesA* and *GH6-1* genes existed in the common ancestor of all extant tunicates.

## 1. Introduction

Horizontal gene transfer (HGT, or lateral gene transfer) is the movement of genetic material between unrelated organisms. Bacterial genomes are greatly shaped by HGT and some of them may contain more than 10% transferred genes [1,2,3]. Although animals usually inherit genetic information from parents [4], many horizontally transferred genes are maintained in animal genomes and expressed [5,6,7]. HGT may well be one of the most important forces shaping animal evolution [5]. One well-recognized example of HGT is the enzyme, tunicate cellulose synthase. Tunicates are the closest living relatives of vertebrates [8,9]. Among animals, tunicates have a unique ability to synthesize and utilize cellulose [10,11,12,13,14]. The tunicate cellulose synthase gene (*CesA*) has an unusual structure. It contains not only a glycosyltransferase (GT2) domain (Glycosyltransferase-like family 2, Pfam PF13641, or CESA_CelA_like, Conserved Domain Database cd06421), but also a Glycosyl Hydrolase family 6 domain (GH6, Pfam: PF01341) [10,11,12,13]. Notably, the GH6 domain of tunicate CesA protein (CesA-GH6) contains an amino acid substitution in the putative active site [10,15] and it may lack hydrolytic activity. A previous analysis of tunicate cellulose synthase failed to identify a cellulose synthase gene in the genome of any other animal [14]. The same analysis also revealed another independent gene (a group of possibly orthologous genes) in tunicate genomes, named *GH6-1*, which contains a GH6 domain [14].

Based on molecular phylogeny and the unique structure of tunicate *CesA*, Nakashima et al. [10] hypothesized that a bacterial genomic region that contained both a *GT2/CesA* gene and a GH6 gene, was transferred horizontally to ancestral tunicates, and that the two genes/domains later merged to form the tunicate *CesA* gene. This hypothesis was further strengthened, when it was observed that actinobacterial genomes contain GC-rich sequences that can be transformed into enhancers in the tunicate cellular environment [16]. Until now, GH6-domain-containing genes have been found in bacteria, fungi, tunicates, and a few other eukaryotes. Because of sequence divergence between tunicate CesA-GH6 domains and GH6-1 proteins, previous studies could not determine the relationship of tunicate CesA-GH6 and GH6-1 proteins with those of other organisms [10,12]. In other words, the evolutionary relationship of the tunicate *GH6-1* gene with other GH6-containing genes remains uncertain.

In eukaryotes, conservation of splice sites (location of boundaries between exons and introns) is often found among orthologous genes [17,18]. Assuming that tunicate GH6-containing genes were transferred horizontally from bacteria, acquisition of spliceosomal introns in tunicate *CesA-GH6* or *GH6-1* genes could be interpreted as a eukaryote-specific character [19,20]. A previous survey [21] found that no splice sites were shared between the tunicate *CesA* genes and plant cellulose synthase genes; therefore, it was concluded that ancient *CesA* genes without introns transferred into tunicate genomes and plant genomes independently.

**Figure 1:**
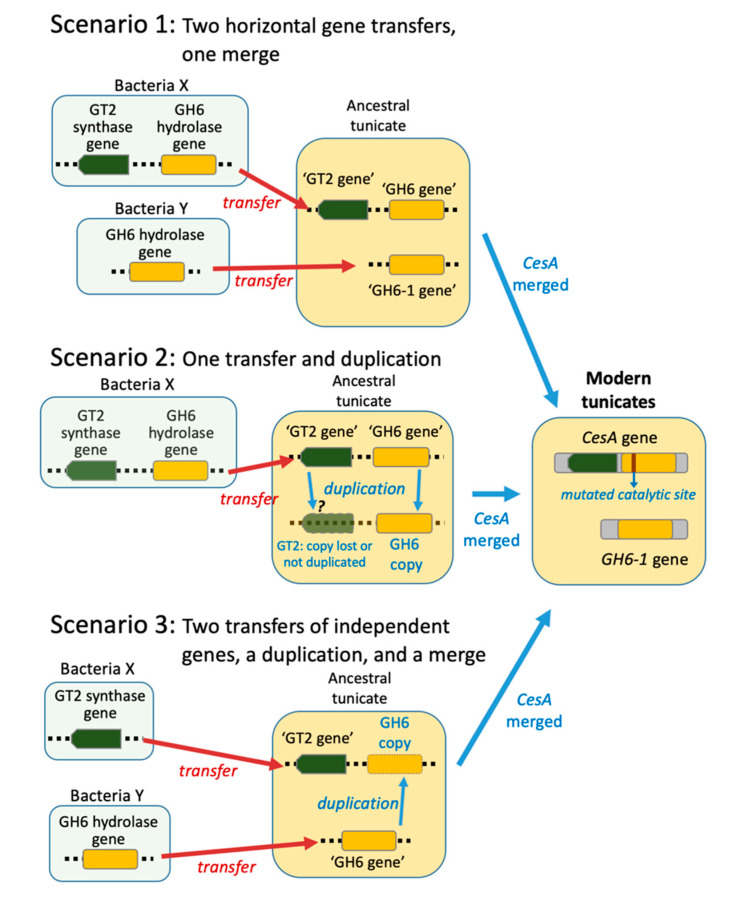
Hypotheses on the origin of tunicate GH6 domain-containing genes. Three scenarios have been proposed to explain the existence of two GH6 domain-containing genes in extant tunicate genomes.

The foregoing finding raised the question of how the tunicate ancestor acquired the precursor of the *CesA-GH6* and *GH6-1* genes. Three possible evolutionary scenarios have been proposed (Figure 1) [10,14]. Scenario 1: Two *GH6* genes were transferred, one of which merged with a GT2-containing gene from the same prokaryote genomic region transferred to an ancestral tunicate and formed the tunicate *CesA* gene. The second GH6-gene gave rise to the current *GH6-1*. Scenario 2: A GH6 gene was transferred and duplicated. After a single transfer of prokaryote GT2-GH6 region into a tunicate genome, a duplication occurred. One copy did not include or retain the GT2 part and became *GH6-1*, while the other copy (an ancient ‘GH6 gene’) merged with the GT2 domain and became part of tunicate *CesA* (joined GT2-GH6 domains). Scenario 3: A GT2-gene and a GH6-gene transferred independently into an ancestral tunicate. The GH6-gene duplicated thereafter. One copy of the GH6 gene fused with the GT2 gene to form the tunicate *CesA* gene. The other copy remained an independent *GH6-1* gene.

In this study, we assessed possible origins of tunicate GH6s by examining phylogenetic relationships of GH6-containing genes in diverse organisms. We also compared sequence characters and exon boundaries among tunicate GH6 domains to understand their evolutionary changes in tunicate genomes.

## 2. Materials and Methods

### 2.1. Re-Analyzing Phylogenetic Relationships of GH6-Containing Proteins of Tunicates, Fungi, Other Eukaryotes, and Bacteria

We reanalyzed genes and gene models of tunicate cellulose synthase (*CesA*) and *GH6-1*, characterized in previous studies (Table 1) [10,11,12,14]. Corresponding gene models and genomic information were retrieved from: NCBI GenBank (reported genes and sequence assemblies: *Salpa thompsoni* genomic assemblies GCA_001749815.1 [22] and transcriptome GFCC00000000.1 [23], and the *Ciona savignyi* transcriptome GGEI00000000.1 [24]), the Ghost database for *Ciona intestinalis* type A (Kyoto University) [25,26,27], the *Botryllus schlosseri* Genome Project (transcripts only, Stanford University) [28,29], the OikoBase for *Oikopleura dioica* [30,31], and the Aniseed database (transcripts and genomes of all other species, as well as the genomes of *C. savignyi* and *B. schlosseri*) [32,33]. (Please note that the name of the species ‘*Ciona intestinalis* type A’ used here follows the name of archived sequence data in databases, including NCBI).

Although the recorded transcripts or annotated gene models were retrieved, we wished to examine whether there is any hidden GH6-encoding genetic information that failed to be annotated as a gene model in each tunicate genome. We first recorded the genomic location (the coordinates on chromosomes, scaffolds, or contigs) of each predicted *GH6-1* and *CesA* gene. When the genomic locations of transcript/models were unknown, as in the cases of *C. intestinalis* type A, *C. savignyi*, *S. thompsoni*, and *O. dioica*, the GH6-containing transcripts were used to search (blastn in BLAST, Basic Local Alignment Search Tool, using default parameters) against its corresponding genome/genomic assembly: the databases used were listed as above. The genomic locations of tunicate GH6-containing genes were listed in Supplementary Table S3. Next, we used the GH6 domains in *C. intestinalis* type A predicted proteins of CesA (GenBank: BAD10864.1) and GH6-1 (NCBI: XP_002119579.1) as queries to search (tblastn in BLAST, with default parameters, e-value threshold = 1×10^−10^) against the other seven tunicates’ genomic database or assemblies and used *O. dioica* predicted proteins (GH6-1, CBY09680.1 and CesA2, BAJ65326.1) to search (tblastn, with default parameters, e-value threshold = 1 × 10^−10^) the *C. intestinalis* type A genome and recorded the genomic locations of results. We found that the location of retrieved transcripts/gene models mostly matched with the BLAST search (tblastn) results, with minor exceptions: a few additional open reading frames (ORF) or short gene models were newly discovered. For example, an ORF of *M. oculata* coding for a 39-amino-acid (AA) peptide and a gene model of *B. schlosseri*, Boschl.CG.Botznik2013.chr9.g44329, coding for an 166-AA peptide, were found in BLAST searches. These short peptides/gene models have similar sequences to a GH6 domain, but those are either far shorter (less than 140 AA) than a typical GH6 domain (Pfam PF01341, with sizes of around 300 AA) or were evaluated as ‘no significance’ in protein profile searches (hmmscan, HmmerWeb version 2.41.1, searched against the Pfam database [34,35]). Therefore, we interpreted that there is no better hidden representative of GH6 genes in these genomes.

We prepared an expanded sequence alignment including more bacterial/fungal GH6 sequences for the phylogenetic analysis. The same two *C. intestinalis* type A protein models (CesA, BAD10864.1 and GH6-1, XP_002119579.1) were used as queries to perform BLAST searches of the NCBI non-redundant protein (nr) database. The blastp (protein-protein BLAST) algorithm was selected, with default parameters (word size = 6; matrix = BLOSUM62; gap cost existence:11, extension:1; conditional compositional score matrix adjustment). A strategy was used to achieve broad sampling of GH6-containing proteins across different taxa. First, the query was used to search all nr sequences excluding tunicates, and the results with the lowest e-values were all sequences from the genus *Streptomyces*. A second search was carried out against “All data excluding tunicates and *Streptomyces.*” Several subsequent searches were performed stepwise, excluding higher taxa (Streptomycetales, Actinobacteria, or Bacteria). Another approach was to search only “Archaea”, “Fungi”, or “Eukaryotes, excluding tunicates and fungi.” A GH6 protein (NCBI: WP_094052291.1) from *Streptomyces* was also used as a query to expand the search results in several eukaryotic taxa (Table 2). However, two questionable ‘eukaryotic’ results, showing higher similarity to bacterial proteins and linkages to other probable bacterial genes, were excluded (Table 2). A few selected bacterial and fungal sequences that were used in a previous phylogenetic analysis [12] were also included in later analyses. In some results, long sequences included conserved domains other than GH6, which were confirmed using InterPro searches (online searches against all available databases) [36]. Those extra domains were excised before downstream analyses. All the selected sequences contained a GH6 domain (Pfam: PF01341), which was confirmed by a hmmscan examination (HmmerWeb version 2.41.1, searched against Pfam database) [34,35]; a GH6 domain in each sequence was identified with an Individual E-value smaller than 1×10^−5^. The multiple sequence alignments were built with MAFFT v7 online server (strategy: L-INS-I iterative refinement recommended for <200 sequences with one conserved domain and long gaps) [37,38]. Poorly aligned regions were removed using trimAl v1.2 [39] when more than 65% of the selected sequences showed gaps in a given position. The appropriate amino acid substitution model was selected using Prottest 3.4.2 (with default parameters) [40] before a maximum likelihood phylogenetic analysis. Phylogenetic reconstructions were performed with MrBayes 3.2.7a (nucmodel = protein, aamodelpr = mixed, ngen = 2,500,000, nchains = 1) [41] or RAxML-HPC Blackbox v8.2.12 (substitution model: PROTCATWAGF, rapid bootstrap with automatic bootstopping) [42] via CIPRES Science Gateway [43]. Consensus trees were visualized with FigTree [44].

### 2.2. Sequence Comparison

Signatures of GH6 proteins were compared with information on PROSITE [45]. Some genes or gene models in the databases had been annotated with exon boundaries. When exon information of genes or gene models was unknown, sequences of transcripts were used to search (blastn, with default parameters) against the corresponding genomic databases: the Ghost database [25,26,27] for *C. intestinalis* type A, NCBI genome assembly GCA_001749815.1 for *S. thompsoni* genomic assembly [22], OikoBase [30,31] for *O. dioica*, and the Aniseed database BLAST tool for other tunicate species [32,33]. Coding parts of transcripts and genomic sequences were then compared with the Splign utility (with default parameters) at NCBI [46]. Tunicate GH6-containing proteins were aligned with MAFFT v7 server (strategy: L-INS-I) [37,38] for splice site (exon-boundary) comparisons.

## 3. Results

### 3.1. Tunicate CesA-GH6 Domains and Tunicate GH6-1 Genes Represent Two Independent Monophyletic Groups

**Figure 2:**
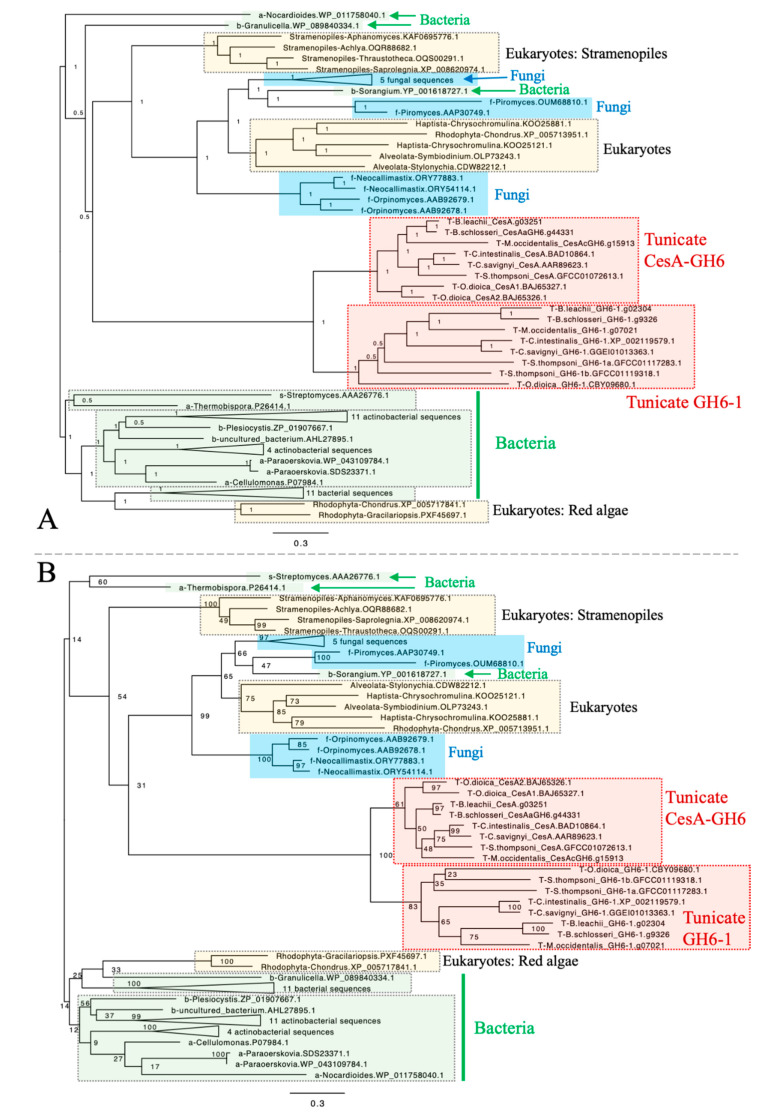
Phylogenetic trees of GH6-containing proteins constructed by Bayesian inference (**A**) and maximum likelihood (**B**). All tunicate sequences formed a cluster. The cluster was further divided into two subclusters of CesA-GH6 domains and GH6-1 proteins. However, the clustering of tunicate GH6 sequences with GH6 proteins of other organisms was not well-supported. Rooting was arbitrary in both panels. Numbers next to internal nodes or branches represent posterior probabilities (in panel **A**) or bootstrap support (in panel **B**) of the neighboring branch. The same trimmed multiple sequence alignment was used as input for both analyses. Bayesian inference was performed with MrBayes using a mixed substitution model (aamodelpr = mixed). The analysis was terminated after 2,500,000 generations as the standard deviation of split frequencies remained as a stable 0.126917 after generation 1,830,000, although this analysis could not reach an ideal convergence due to short sequence lengths and divergent data. The maximum likelihood analysis was performed with RAxML-HPC BlackBox on CIPRES Science Gateway. The WAG amino acid substitution model with empirical base frequencies was selected and bootstrapping was automatically stopped after 804 cycles. The starting part of sequence names represents its source organism category, in alphabetical order: a, Actinobacteria, excluding *Streptomyces*; b, Bacteria excluding Actinobacteria; f, fungi; s, genus *Streptomyces*; T, tunicates. Fully-expanded trees are shown as supplementary figures. Scales represent expected changes per site.

To determine whether *GH6-1* genes represent a monophyletic group distinct from tunicate *CesA* genes and to understand the relationship of GH6-1 with GH6 proteins in other organisms, we used amino acid sequences of eight predicted tunicate CesA-GH6 domains, sequences of eight predicted GH6-1 proteins, and many predicted GH6 protein sequences from bacteria, fungi, and various eukaryotes (Supplementary Table S1) to reconstruct phylogenetic trees (Figure 2, Supplementary Figures S1 and S2). Both Bayesian inference (Figure 2A) and maximum likelihood (ML) (Figure 2B) approaches provided trees supporting a close relationship of tunicate CesA-GH6 and GH6-1. In addition, CesA-GH6 sequences and GH6-1 sequences formed two separate clusters, although the ML bootstrap support values were only 83% for the GH6-1 clade and 61% for the CesA-GH6 clade.

### 3.2. The Origin of Tunicate GH6 Domains Is Hard to Deduce

Nonetheless, our analyses failed to determine the relationship of tunicate sequences among other GH6 proteins. Although in these trees, tunicate sequences were clustered with many fungal GH6 proteins, some other eukaryotic GH6s (from red algae (Rhodophyta), Haptista, and the SAR supergroup), and a proteobacterial GH6 (YP_001618727.1, *Sorangium cellulosum*), the Bayesian posterior probability (Figure 2A) and ML bootstrap support (Figure 2B) for this clustering were low. Notably, branches leading to tunicate sequences were longer than branches to other sequence clusters.

### 3.3. Many Tunicate GH6-1 Proteins Maintain the Probable Active Site, in Contrast to Tunicate CesA Proteins

**Figure 3:**
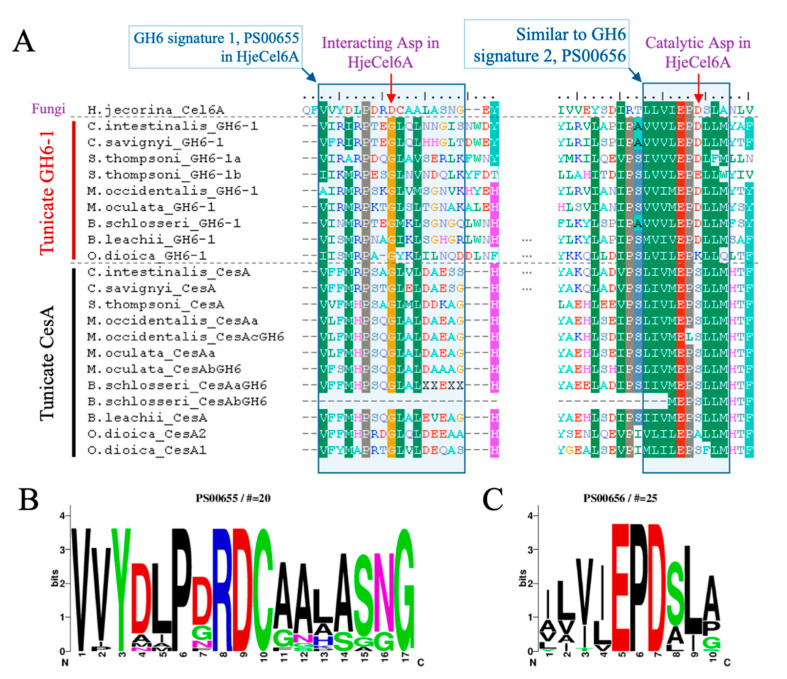
Amino acid conservation of tunicate GH6-domain-containing proteins. (**A**) GH6-1 proteins from ascidians and the GH6-1a from *Salpa thompsoni* have aspartic acids that correspond to the catalytic center of fungal Cel6A protein; however, another *S. thompsoni* GH6-1 protein (SthGH6-1b), an *Oikopleura* GH6-1 protein, and tunicate CesA proteins show other amino acids at this site. Similar amino acids under the BLOSUM62 matrix are color-shaded. HjeCel6A: *H. jecorina* Exoglucanase 2, UniProtKB P07987.1. (**B–C**) Sequence logos of Glycosyl Hydrolases Family 6 Signature 1 (PROSITE entry PS00655, panel **B**) and Glycosyl Hydrolases Family 6 Signature 2 (PROSITE entry PS00656, panel **C**), showing the amino acid frequency of each site.

With the sequence alignment of tunicate GH6-1 and CesA proteins, we compared their sequence signatures to those of other GH6 proteins. The enzymatic activity of Exoglucanase 2 (Cel6A) of *Hypocrea jecorina* (formerly *Trichoderma reesei*) was well characterized [15]. The aspartic acid at position 221 of *H. jecorina* Cel6A (Hje-D221) serves as the catalytic center [15]. We found that in many tunicate GH6-1 proteins, an aspartic acid can be aligned to the catalytic *H. jecorina* D221 (Figure 3A), except for SthGH6-1b (E197) and OdiGH6-1 (K211). However, the catalytic aspartic acid was not conserved in tunicate CesA (Figure 3A). Tunicate proteins also show a sequence environment that almost matches (8–9 out of 10 amino acids) the conserved ‘signature 2’ of GH6 (Figure 3C: PROSITE PS00656: [LIVMYA]-[LIVA]-[LIVT]-[LIV]-E-P-D-[SAL]-[LI]-[PSAG]).

Another signature of GH6 that also contributes to catalytic ability is PS00655 (Figure 3B, including another important aspartic acid, D175, in the *H. jecorina* protein [15]), but this signature was not conserved in tunicate GH6-containing proteins. In the aligned region, ≤40% of amino acids matched the signature pattern.

### 3.4. Splice Site Conservation in Tunicate CesA Genes or GH6-1 Genes

We arranged the positions of coding exon splice sites (exon boundaries) of tunicate *GH6-1* and the GH6 domain of *CesA* genes and then registered all sites to an aligned amino acid sequence matrix for comparison. For example, the splice site V217.frame+2 of CinGH6-1 means that the last nucleotide of an exon locates at the *second* codon position for amino acid 217 (valine) of *C. intestinalis* type A GH6-1 protein. Similarly, the site K316.frame+3 means that the last nucleotide of an exon is the nucleotide of the *third* codon position for amino acid 316 (lysine).

Several splice sites matched among tunicate GH6-1 proteins (Table 3), and these matching splice sites also have the same frame as the exon-intron boundary. Therefore, we consider them genuine shared splice sites. For example, the site Cin316 was shared by eight *GH6-1* genes from seven tunicate species.

An obscure case was that of the *O. dioica CesA1* R1100 site. Although one *O. dioica* exon boundary was located in a codon for an arginine that could be aligned to the amino acids of splice site Cin316 of GH6-1 proteins, the location of the splice site was shifted by one nucleotide. Our results do not indicate that *O. dioica CesA1* shares this splice site with tunicate *GH6-1* genes.

Excluding the foregoing case, we found no splice site shared between tunicate GH6-1 and CesA. However, several other splice sites are shared within CesA protein GH6 domains (Supplementary Table S2).

### 3.5. Genomic Locations of Tunicate CesA Genes and GH6-1 Genes are Separated

During the comparison of tunicate *GH6-1* and *CesA-GH6* transcripts to genomic DNA, we noted that most *GH6-1* genes and *CesA-GH6* genes are located separately in the genome (Supplementary Table S3). In *C. intestinalis* type A, for which a chromosome-level genome is available [47], the *GH6-1* and *CesA* genes are located in chromosomes 3 and 7, respectively. Although gene models of a GH6-1 and a GH6-2 appear on chromosome 9 of *Botryllus schlosseri*, these two gene models are separated by about 2.65 million base pairs. In other species, although draft genomes are in scaffold-level assemblies, *GH6-1* and *CesA-GH6* do not appear on the same scaffold/contig. These results suggest a reduced likelihood of tandem duplication of an ancestral *GH6* gene.

## 4. Discussion

### 4.1. Two GH6-Containing Genes Exist in Tunicate Genomes

In this study, we first tried to resolve the relationship of a recently discovered tunicate GH6-containing gene (*GH6-1*), the GH6 part of the tunicate *CesA* gene (called *CesA-GH6*), and GH6-containing genes from other organisms. The result was that tunicate CesA-GH6 and GH6-1 sequences form two clusters (Figure 2), indicating that these are two monophyletic groups and that both were inherited from the tunicate common ancestor. On the other hand, in phylogenetic reconstructions, the grouping of tunicate GH6-containing proteins and other GH6s was not conclusive (Figure 2). There were long branches that thwarted conclusive results regarding the relationship of tunicate GH6-containing proteins and those of other organisms. We suspect that the highly evolved tunicate GH6-containing-proteins cause long-branch attraction, adversely affecting tree topologies. Based on current phylogenetic trees, we could not confidently propose a non-tunicate GH6 protein(s) that represents the closest relative(s) to tunicate GH6-containing proteins. Considering branch lengths and the tree topology of GH6 proteins, it is possible that an ancient GH6 gene evolved highly, soon after it was transferred into an ancestral tunicate. After the transfer event, this GH6 gene likely duplicated in the tunicate genome. We drew this conclusion because of clustering of tunicate CesA-GH6 and GH6-1 groups, in which no genes of other organisms were inserted. Therefore, either scenario 2 or 3 in Figure 1 could explain the origin of tunicate GH6-containing genes. However, as we could not propose a candidate donor species/lineage of tunicate GH6s, we cannot directly evaluate the two possible scenarios further.

Assuming that tunicate GH6-genes were acquired via HGT event(s), no other tunicate genes would help to resolve the current, ambiguous tree topology. On the other hand, it is intriguing that many, but not all, GH6 proteins from other eukaryotes (including GH6s of fungi, the SAR supergroup, Haptista, and red algae) were clustered close to tunicate CesA-GH6. Recently, it was shown that some fungi retain many genes acquired from bacteria [48]. Therefore, future disclosures of eukaryotic genes similar to tunicate GH6 genes may provide important information on possible horizontal gene transfer events. As we found no GH6 genes in Archaea (Table 2), GH6 genes may have been transferred from bacteria to multiple eukaryotes in parallel. Alternatively, GH6 genes could also have been transferred between different eukaryotic organisms.

The separate genomic locations of tunicate *CesA* genes and *GH6-1* genes (Supplementary Table S3) imply that the two genes did not stem from recent tandem duplication events, so these genes have been regulated in different genomic contexts.

### 4.2. Lineage-Specific Gene Content Change Along with Sequence Signature Conservation

We found multiple transcripts or gene models representing *GH6-1* (or multiple CesA-GH6s) in the genomes of some tunicate species (Table 1 and Supplementary Table S3). Some of them may represent true lineage-specific duplications, as in the case of the two *CesA* genes of *O. dioica* [12]. For example, the two *S. thompsoni* GH6-1 proteins have only 35% identical amino acids when aligned and compared. They also showed long terminal branches in phylogenetic trees. In addition, although the current *S. thompsoni* genome had been assembled into sub-chromosome level scaffolds, these two *GH6-1* genes corresponded to different genomic scaffolds. However, some gene models and open reading frames are highly similar to (around 90% amino acid identity) and shorter than another gene model in the same genome. For example, one GH6-1 protein model of *B. schlosseri* (BscGH6-1b, g61144, chromosome unassigned) showed 97.5% identity to BscGH6-1 (g9326, chromosome 9). These could be more recently duplicated genes. Alternatively, these may just be different alleles annotated separately due to imperfections of software-based genome assembly and may not represent a true species-specific duplication. Some gene models contain the GH6 part, but not the CesA/GT2 part of the tunicate *CesA* gene. Based on our knowledge that a typical, complete tunicate *CesA* gene contains a CesA/GT2 part and a GH6 part, it is possible that the CesA/GT2 part of a complete tunicate *CesA* gene was erroneously predicted as another gene model in the aforementioned cases, similar to a previous observation on a sea urchin genome [49] and several amphioxus gene models [50]. We also found that one *CesA* model of *M. oculata* (*MocCesAa*, Moocul.CG.ELv1_2. S71617.g04842.01.t) is obviously larger. It also encodes a rhodopsin-like G protein-coupled receptor domain (Interpro: IPR000276) at its upstream end. It would require further studies to confirm whether it is a true merged gene, a mistake in genome assembly and annotation, or a polycistronic operon, similar to those of *O. dioica* or *C. intestinalis* type A [51,52].

This analysis of GH6-1 and CesA-GH6 sequence signatures shows that, although both tunicate GH6-1 proteins and CesA-GH6 domains contain a region that almost matches the conserved GH6-signature 2 (PROSITE PS00656), the probable catalytic aspartic acid exists only in GH6-1 proteins and not in CesA-GH6. This aspartic acid is conserved in most non-tunicate GH6 proteins (56 out of 58 sequences compared in this study). Mutation of this possible catalytic site in *CesA* genes probably occurred very early in an ancestral tunicate before the branching of the larvacean (Appendicularia) clade. Despite the loss of the aspartic acid, the conservation of other amino acids at the signature site may imply that this domain acquired novel function in tunicates. Nevertheless, whether tunicate GH6-1 proteins or CesA-GH6 domains possess any catalytic activity remains to be determined.

### 4.3. Shared Splice Sites Indicate the Ancient History of Tunicate GH6 Genes

In this study, we found several shared splice sites among tunicate *GH6-1* genes. We also extended the comparison of shared splice sites of *CesA* genes to other tunicate species. As previously reported [12], 17 splice sites in *CesA* genes of *C. intestinalis* type A and *C. savignyi* are still conserved after about 100 million years of independent evolution [53]. In addition, a splice site shared by *CesA2* of *O. dioica* and *O. longicauda*, *CesA* of *Halocynthia roretzi*, *Molgula tectiformis*, and two *Ciona* species was interpreted as support for common ancestry of all tunicate *CesA* genes [13]. In this study, although we found no other sites shared between genes of *O. dioica* and other tunicates, we found that many shared splice sites are present among GH6-containing genes from three other major clades of tunicates (Thaliacea + Phlebobranchia + Stolidobranchia). It is reasonable to assume that many shared introns were acquired after the branching of larvaceans and before the subsequent divergence of major tunicate clades.

There was no well-supported splice site shared between *GH6-1* and *CesA-GH6*. Assuming that only one *GH6* gene was transferred horizontally into an ancestral tunicate genome, the lack of shared splice sites between *GH6-1* and *CesA-GH6* may indicate that the ancient *GH6* gene had no introns when it was transferred into the tunicate genome. This supports a previous interpretation about the *CesA* transfer event [21].

The obscure *O. dioica CesA1* splice site (R1100) differs by just one nucleotide from the Cin316 splice site of *GH6-1* genes. It may simply have resulted from an independent intron acquisition event. Alternatively, this could represent a shared splice site that experienced a one-nucleotide intron shift [54], but this requires further investigation. Moreover, no other *CesA* genes we examined show a splice site here. If the GH6 part of the ancient *CesA* gene contained that intron, other *CesA* genes must have undergone intron loss. Therefore, it is not a parsimonious explanation.

The presence of two *CesA* genes in *O. dioica* raised another question of whether tunicate *CesA* was duplicated before larvaceans diverged [12] (see also Figure 2). The observation that *Ciona CesA* genes share a splice site with *OdiCesA2*, but not *OdiCesA1*, may favor the scenario of early duplication [12]. In our analysis, the splice site discussed previously, Cin976, was found in the *OdiCesA2* and *CesA* genes of at least six other tunicate species (Supplementary Table S2), but this splice site was not found in *M. oculata*. Therefore, it is possible that *O. dioica* had a lineage-specific duplication of the *CesA* gene and that one copy (*CesA1*) lost this intron.

### 4.4. Future Perspective

It is likely that a GH6-containing gene was transferred to and duplicated in ancient tunicate genomes before major tunicate lineages diverged. The two tunicate GH6-containing genes acquired different introns and have preserved part of that sequence signature. We anticipate that future larvacean transcriptomic studies that are complementary to recent larvacean genome projects (for example, [55]) will provide a better understanding of tunicate GH6-containing genes and tunicate genome evolution.

In plants, activity of cellulase is required to regulate cellulose synthesis and growth of cell walls [56]. Therefore, it is important to examine whether any hydrolase activity of tunicate GH6-containing proteins could also influence cellulose synthesis and physiology. One approach would be to examine enzymatic activity in vitro, and another would be to genetically manipulate animals using genome-editing methods [57,58]. These are subjects for future studies.

## Figures and Tables

**Table 1:** Tunicate GH6-containing genes or gene models and related genes analyzed in this study.

Species	Domain Content	Short Name of the Gene Used in this Manuscript *	Source Database	Accession/ID of Gene, Transcript, or Protein	Note
*Ciona intestinalis* type A *(C. robusta)*	GH6	*CinGH6-1*	GenBank	XM_002119543.4/XP_002119579.1	
	CesA+GH6	*CinCesA*	GenBank	NM_001047983.1/BAD10864.1	As reported in [10]
*Ciona savignyi*	GH6	*CsaGH6-1*	GenBank (Transcriptome)	GGEI01013363.1	
	CesA+GH6	*CsaCesA*	GenBank	AY504665.1/AAR89623.1	As reported in [11]
*Salpa thompsoni*	GH6	*SthGH6-1a*	GenBank (Transcriptome)	GFCC01117283.1	Possible lineage-specific duplication
	GH6	*SthGH6-1b*	GenBank (Transcriptome)	GFCC01119318.1	No possible catalytic Asp; possible lineage-specific duplication.
	CesA+GH6	*SthCesA*	GenBank (Transcriptome)	GFCC01072613.1	
*Molgula occidentalis*	GH6	*MoxGH6-1*	Aniseed database	Moocci.CG.ELv1_2.S285391.g07021.01.t	
	CesA+GH6	*MoxCesAa*	Aniseed database	Moocci.CG.ELv1_2.S469068.g15915.01.t	Short GH6 part
	GH6	*(MoxCesAbGH6)*	Aniseed database	Moocci.CG.ELv1_2.S469068.g15914.01.t	Very short
	GH6	*MoxCesAcGH6*	Aniseed database	Moocci.CG.ELv1_2.S469068.g15913.01.t	
*Molgula oculata*	GH6	*MocGH6-1*	Aniseed database	Moocul.CG.ELv1_2.S112948.g12660.01.t	
	CesA+GH6	*MocCesAa*	Aniseed database	Moocul.CG.ELv1_2.S71617.g04842.01.t	Rhodopsin-like GPCR domain at upstream part
	GH6	*MocCesAbGH6*	Aniseed database	Moocul.CG.ELv1_2.S69739.g04625.01.t	
*Botryllus schlosseri*	GH6	*BscGH6-1*	*Botryllus schlosseri* Genome Project	g9326	
	GH6	*(BscGH6-1b)*	*Botryllus schlosseri* Genome Project	g61144	Short, similar to BscGH6-1
	GH6	*BscCesAaGH6*	*Botryllus schlosseri* Genome Project	g44331	Similar to BscCesAbGH6 (89.6% identity in the matching 222 AA region)
	GH6	*BscCesAbGH6*	*Botryllus schlosseri* Genome Project	g45080	Similar to BscCesAaGH6
*Botrylloides leachii*	GH6	*BleGH6-1*	Aniseed database	Boleac.CG.SB_v3.S133.g02304.01.t	
	CesA+GH6	*BleCesA*	Aniseed database	Boleac.CG.SB_v3.S157.g03251.01.t	
*Oikopleura dioica*	GH6	*OdiGH6-1*	OikoBase/GenBank	GSOIDT00010490001/CBY09680.1	
	GH6	*(OdiGH6-1b)*	OikoBase/GenBank	GSOIDT00021901001/CBY33927.1	98% identical to OdiGH6
	CesA+GH6	*OdiCesA2*	GenBank	AB543593.1/BAJ65326.1	As reported in [12,13]
	CesA+GH6	*OdiCesA1*	GenBank	AB543594.1/BAJ65327.1	As reported in [12,13]

* Gene names were assigned after considering phylogenetic information examined in this study and in that by Inoue et al. [14].

**Table 2:** GH6 proteins in different taxa.

Taxa				GH6 presence?
Bacteria				**Present**
Archaea				Not yet observed
Eukaryota	Opisthokonta	Metazoa	tunicates	**Present**
			Metazoa, except tunicate	No? Contamination? *1
		Fungi		**Present**
		Opisthokonta, except Metazoa and fungi		Not yet observed
	Viridiplantae			No? Contamination? *2
	SAR-Stramenopiles			**Present**
	SAR-Alveolate			**Present**
	SAR-Rhizaria			Not yet observed
	Haptista			**Present**
	Rhodophyta			**Present**
	Other eukaryotes			Not yet observed

*1: A GH6 protein in the *Lucilia cuprina* (a dipteran) genome project, XP_023300643.1, was very similar to bacterial GH6 proteins. It was located at a genomic scaffold that contained other probable bacterial genes. *2: A GH6 protein found in the *Gossypium hirsutum* (upland cotton) genome project, XP_016733546.1, was highly similar to bacterial GH6 proteins and it was located at a genomic scaffold that contained other probable bacterial genes. The above two cases were the only results that contained GH6 domains in each search. We treated these two cases as bacterial contaminants.

**Table 3:** Splice site matches of tunicate GH6-1 proteins.

		Splice site name		
		Cin217	Cin256	Cin316
**Protein**	**Introns within coding region**	**Splice site residue & frame**		
CinGH6-1	3	V217, +2	G256, +1	K316, +3
CsaGH6-1	3	V223, +2	G262, +1	K322, +3
SthGH6-1a	6	E229, +2	G268, +1	P328, +3
SthGH6-1b	5	K230, +2	G269, +1	K329, +3
MoxGH6-1	3	R222, +2	G260, +1	A320, +3
MocGH6-1	2	n.s.*^1^ (R222)	G260, +1	A320, +3
BscGH6-1	5	K335, +2	G373, +1	A433, +3
BleGH6-1	4	K229, +2	n.s.*^2^ (G285)	A345, +3
OdiGH6-1	6	n.s.*^1^ (N244)	n.s.*^2^ (G282)	n.s.*^1^ (K343)
OdiCesA1*^3^	8	n.s.*^1^ (R1001)	n.s.*^2^ (G1040)	R1100*^3^, frame +2

All matching splice sites found in this study are C-terminal to the possible catalytic center: positions 178–187 in *C. intestinalis* type A GH6-1. *^1^: No splice (n.s.) site at the aligned amino acid and the amino acid is not conserved; *^2^: No splice (n.s.) site at the aligned amino acid, although this position encodes a conserved glycine; *^3^: The splice site OdiCesA1-R1100 could be aligned with splice site Cin316 of GH6-1 proteins at the amino acid level, but there is a one-nucleotide position difference and it may not represent a shared splice site.

## Supplementary Materials

The following are available online at https://www.mdpi.com/2073-4425/11/8/937/s1, Supplementary Table S1. Other GH6-containing proteins used in phylogenetic analyses. Supplementary Table S2. Shared splice sites in the GH6 domain of tunicate CesA proteins. Supplementary Table S3. Tunicate GH6-containing genes or gene models and their genomic locations. Supplementary Figure S1: The fully expanded phylogenetic tree of Figure 2A, showing Bayesian inference of phylogenetic relationships of GH6 proteins. Supplementary Figure S2: The fully expanded phylogenetic tree of Figure 2B, showing a maximum likelihood reconstruction of phylogenetic relationships of GH6 proteins.
