# Supplementary material for: Phylogenetic Analyses of Glycosyl Hydrolase Family 6 Genes in Tunicates: Possible Horizontal Transfer
Source: Genes (Basel). 2020 Aug 13;11(8):937. doi: 10.3390/genes11080937 (PMC7464555; doi:10.3390/genes11080937)
Supplement: Supplementary file 1 [file genes-11-00937-s001.pdf]

Supplementary Table S1. Other GH6-containing proteins used in phylogenetic analyses.

Taxa	(taxa-) Genus	Accession Number
**Actinobacteria, except *Streptomyces***	unidentified Actinobacteria	WP_054220290.1
	Actinoplanes	WP_043523455.1
	Actinoplanes	WP_043525061.1
	Brachybacterium	WP_012804931.1
	Cellulomonas	P07984.1
	Geodermatophilus	PYG45136.1
	Geodermatophilus	WP_163476570.1
	Kitasatospora	WP_030394430.1
	Mycobacteriaceae	WP_011562673.1
	Mycobacterium	YP_001848433.1
	Mycobacterium	YP_879613.1
	Mycolicibacterium	WP_005142664.1
	Nocardioides	WP_011758040.1
	Paraoerskovia	SDS23371.1
	Paraoerskovia	WP_043109784.1
	Saccharopolyspora	WP_009943607.1
	Streptacidiphilus	WP_034091765.1
	Thermobispora	P26414.1
	Zhihengliuella	WP_130448960.1
**Bacteria, except Actinobacteria**	*Cystobacter*	WP_095990604
	Granulicella	WP_089840334.1
	Myxococcus	WP_090493059.1
	Myxococcus	WP_140855929.1
	Plesiocystis	ZP_01907667.1
	Sorangium	YP_001618727.1
	Uncultured bacterium	AHL27895.1
	Vitiosangium	WP_108069950.1
**Eukaryotes, except fungi and tunicates**	Alveolata- *Stylonychia*	CDW82212.1
	Alveolata- *Symbiodinium*	OLP73243.1
	Haptista- *Chrysochromulina*	KOO25121.1
	Haptista- *Chrysochromulina*	KOO25881.1
	Rhodophyta- *Chondrus*	XP_005713951.1
	Rhodophyta- *Chondrus*	XP_005717841.1
	Rhodophyta- *Gracilariopsis*	PXF45697.1
	Stramenopiles- *Achlya*	OQR88682.1
	Stramenopiles- *Aphanomyces*	KAF0695776.1
	Stramenopiles- *Saprolegnia*	XP_008620974.1
	Stramenopiles- *Thraustotheca*	OQS00291.1
**Fungi**	*Neocallimastix*	ORY54114.1
	Neocallimastix	ORY77883.1
	Orpinomyces	AAB92678
	Orpinomyces	AAB92679.1
	Piromyces	AAP30749.1
	Piromyces	OUM68810.1
	Talaromyces	APE61639.1
	Talaromyces	ATQ35966.1
	Talaromyces	BAA74458.1
	Termitomyces	KNZ78466.1
	Volvariella	AAT64008.1
***Streptomyces* (genus)**	*Streptomyces*	AAA26776.1
	Streptomyces	WP_033308106.1
	Streptomyces	WP_079662022.1
	Streptomyces	WP_093802936.1
	Streptomyces	WP_094052291.1
	Streptomyces	WP_099499477.1
	Streptomyces	WP_120721336.1
	Streptomyces	WP_150216144.1
	Streptomyces	WP_156692247.1

Supplementary Table S2. Shared splice sites in the GH6 domain of tunicate CesA proteins.

	Splice site name			
	Cin853	Cin930	Cin976 *1	Cin1070
Protein	Splice site residue & frame			
CinCesA	G853, +1	A930, +1	Q976, +3	R1070, +2
CsaCesA	G1137, +1	E1214, +1	Q1260, +3	R1354, +2
SthCesA	G870, +1	E947, +1	Q993, +3	M1087, +2
MoxCesAa	G938, +1	n.s.*2 (G1015)	gap *3	gap
MoxCesAcGH6	G38, +1	n.s. (G115)	L161, +3	R255, +2
MocCesAa	n.s. (G1505)	n.s. (G1582)	n.s. (Q1628)	R1722, +2
MocCesAbGH6	n.s. (G33)	n.s. (G110)	n.s. (Q156)	R250, +2
BscCesAaGH6	n.s. (G2)	G79, +1	Q125, +3	R219, +2
BscCesAbGH6	gap	gap	Q22, +3	R116, +2
BleCesA	G946, +1	G1023, +1	Q1069, +3	R1163, +2
OdiCesA2	n.s. (G848)	n.s. (E925)	Q971, +3	n.s. (R1065)
OdiCesA1	n.s. (G866)	n.s. (P943)	n.s. (Q989)	n.s. (R1083)

*1: This shared splice site has been reported in previous studies [12,13] and used to infer evolutionary relationships of tunicate *CesA* genes. *2 n.s.: No splice site at this position/codon. *3 gap: A gap in this sequence in multiple alignment, possibly representing deletion of a segment, or that the gene model is not complete.

Supplementary Table S3. Tunicate GH6-containing genes or gene models and their genomic locations.

**Species, Genome version**	**Domain content**	**Short name of the gene used in this manuscript**	**Accession/ID of gene or transcript**	**Chromosome or genomic scaffold location**
Ciona intestinalis type A *(C. robusta)*, HT Reference Genome (2019 version, Ghost database)	GH6	*CinGH6-1*	XM_002119543.4	Chr3: 2879389-2883393
	CesA+GH6	*CinCesA*	NM_001047983.1	Chr7: 3393711-3406917
Ciona savignyi, Ciona_savignyi_ENS81_Genome (Aniseed database)	GH6	*CsaGH6-1*	GGEI01013363.1	Reftig R35: 1310928-1318413
	CesA+GH6	*CsaCesA*	AY504665.1	Reftig R2: 2893501-2913534
Salpa thompsoni, GenBank assembly: GCA_001749815.1 (NCBI)	GH6	*SthGH6-1a*	GFCC01117283.1	Scaffolds: 3051, 10336, and 13318
	GH6	*SthGH6-1b*	GFCC01119318.1	Scaffolds: 26886, contig 211572 and 272169
	CesA+GH6	*SthCesA*	GFCC01072613.1	Scaffolds: 5822, 14051, 39468, 41682, 48268
Molgula occidentalis, Molgula_occidentalis_ELv12_Genome (Aniseed database)	GH6	*MoxGH6-1*	Moocci.CG.ELv1_2.S285391.g07021.01.t	Scaffold 285391: 1115-4105
	CesA+GH6	*MoxCesAa*	Moocci.CG.ELv1_2.S469068.g15915.01.t	Scaffold 469068: 7725-12872
	GH6	*(MoxCesAbGH6)*	Moocci.CG.ELv1_2.S469068.g15914.01.t	Scaffold 469068: 6382-7088
	GH6	*MoxCesAcGH6*	Moocci.CG.ELv1_2.S469068.g15913.01.t	Scaffold 469068: 2541-4008
Molgula oculata, Molgula_oculata_ELv12_Genome (Aniseed database)	GH6	*MocGH6-1*	Moocul.CG.ELv1_2.S112948.g12660.01.t	Scaffold 112948: 22155-24431
	CesA+GH6	*MocCesAa*	Moocul.CG.ELv1_2.S71617.g04842.01.t	Scaffold 71617: 7004-16648
	GH6	*MocCesAbGH6*	Moocul.CG.ELv1_2.S69739.g04625.01.t	Scaffold 69739: 3301-4739
Botryllus schlosseri, Botryllus_schlosseri_botznik2013_Genome (Aniseed database); *Botryllus schlosseri* Genome Project (transcript GFF information, Stanford University)	GH6	*BscGH6-1*	g9326/Boschl.CG.Botznik2013.chr9.g09326.01.t	Chr9: 18,466,231-18,474,930
	GH6	*(BscGH6-1b)*	g61144 (only in the genome project transcript fasta/GFF)	Contig botctg111009: 2832-3434
	GH6	*BscCesAaGH6*	g44331/Boschl.CG.Botznik2013.chr9.g44331.01.t	Chr9: 15,780,634 -15,782,886
	GH6	*BscCesAbGH6*	g45080/Boschl.CG.Botznik2013.chr13.g45080.01.t	Chr13: 9,500,441-9,502,510
Botrylloides leachii, Botrylloides_leachii_SBv3_Genome (Aniseed database)	GH6	*BleGH6-1*	Boleac.CG.SB_v3.S133.g02304.01.t	Scaffold 133: 25041-28636
	CesA+GH6	*BleCesA*	Boleac.CG.SB_v3.S157.g03251.01.t	Scaffold S157: 172941-185066
Oikopleura dioica, Odioica_Assembly_reference_unmasked_v3.0 (OikoBase)	GH6	*OdiGH6-1*	GSOIDT00010490001	Scaffold 33: 182048-186143
	GH6	*(OdiGH6-1b)*	GSOIDT00021901001	Scaffold 33: 182048-185894
	CesA+GH6	*OdiCesA2*	AB543593.1	Scaffold 314: 1902-7896
	CesA+GH6	*OdiCesA1*	AB543594.1	Scaffold 80: 135240-139503

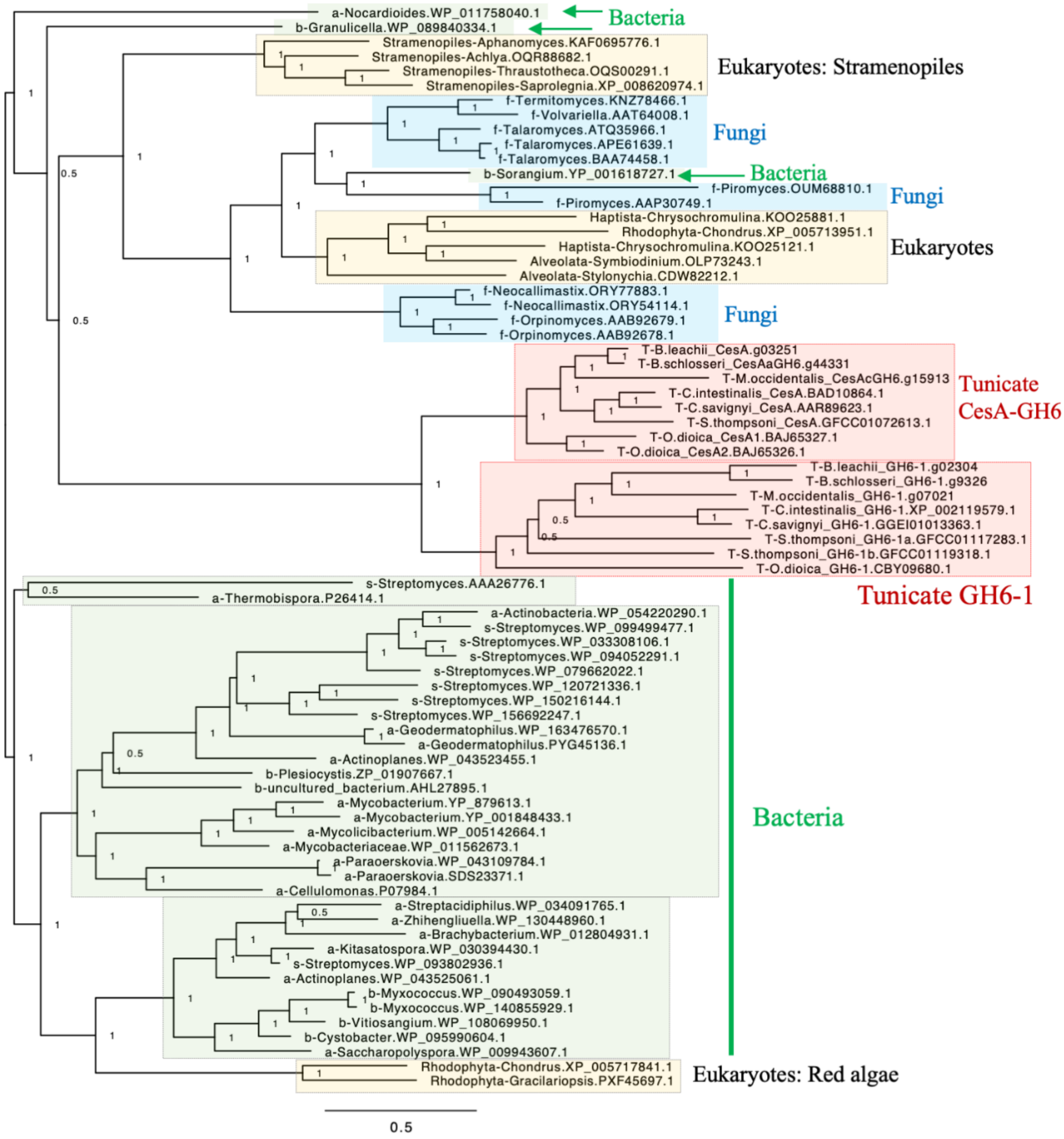

Supplementary Figure S1: The fully expanded phylogenetic tree of Figure 2A, showing Bayesian inference of phylogenetic relationships of GH6 proteins.

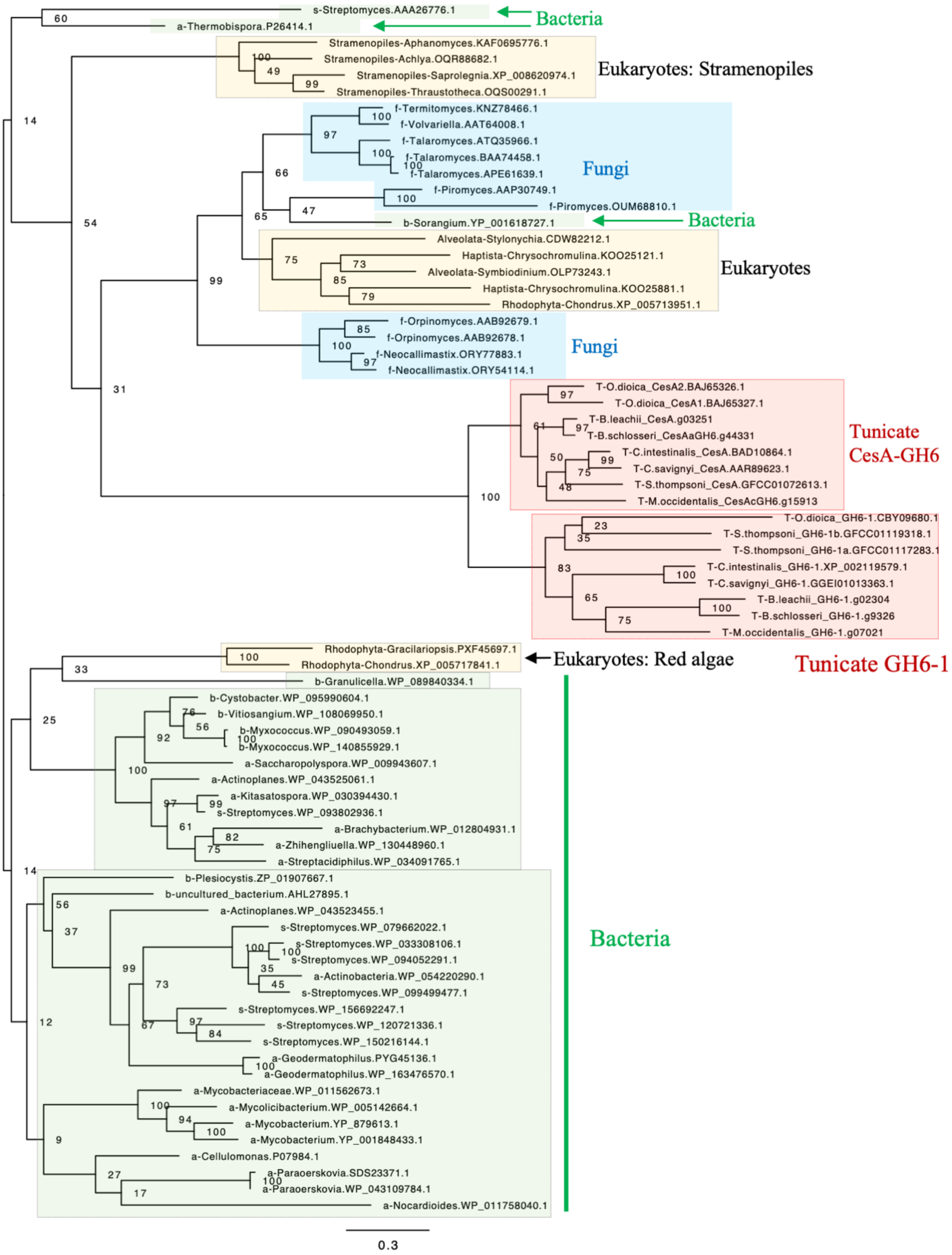

Supplementary Figure S2: The fully expanded phylogenetic tree of Figure 2B, showing a maximum likelihood reconstruction of phylogenetic relationships of GH6 proteins.
